# Perceived usefulness of open educational resources: Impact of switching to online learning for face-to-face and distance learners

**DOI:** 10.3389/fpsyg.2022.1004459

**Published:** 2023-01-18

**Authors:** Simon K. S. Cheung, Billy T. M. Wong, Kam Cheong Li

**Affiliations:** ^1^Information Technology Office, Hong Kong Metropolitan University, Hong Kong, China; ^2^Office of Research Affairs, Hong Kong Metropolitan University, Hong Kong, China; ^3^School of Open Learning, Hong Kong Metropolitan University, Hong Kong, China

**Keywords:** open educational resources, online learning, face-to-face learner, distance learner, COVID-19

## Abstract

This paper reports a study on the perceived usefulness of university students on open educational resources (OER) in relation to the switch of learning mode to online learning during the COVID-19 pandemic. The participants involved two groups of students, one studying in a face-to-face mode and the other in a distance learning mode. They took part in a survey which was conducted in 2019 before the pandemic (with a total of 912 responses) and 2021 during the pandemic (with a total of 1,018 responses). The results show that both groups of students generally perceived OER to be more useful during the pandemic. The specific types of OER which were perceived as relatively more useful include open online courses and open access textbooks. Face-to-face students showed a higher level of perceived usefulness of OER for preparing tests and examinations, while distance learning students perceived OER as more useful for supplementing course materials. They both concerned about the limitations of OER, especially on accuracy and comprehensiveness. The findings suggest the importance of recognizing the diverse needs of the two groups of students and offering appropriate OER support for them.

## 1. Introduction

Since their development in the early 2000s, open educational resources (OER) have become increasingly prevalent not only as an open source of learning resources but also as an agent to help transform teaching and learning with an aim to make learning more accessible and equitable (Miao et al., 2016). OER are regarded as teaching and learning resources released under an open license and are allowed for reuse, revision, and redistribution with no or limited restrictions (Li and Wong, 2021). Relevant resources have been well available for higher education. Popular types of resources cover open courseware and course materials, open online courses and tutorials, open e-books and e-journals, and open-source learning tools (Cheung et al., 2013; Blomgren, 2018; Wong and Li, 2019).

The use of OER has shown to bring a broad range of benefits for learners. It helps learners to access and use learning materials easily, reduce their cost and dismantle learning barriers, facilitate sharing of learning materials and collaboration, and improve learning performance. The extent to which learners can benefit from OER depends on factors such as the availability of support, learners’ computer literacy, and availability of suitable resources (Li and Wong, 2015).

Learners’ perception on the usefulness of OER is one of the major factors affecting their intention and behavior of engaging in OER (Wong et al., 2016). There have been a series of studies conducted in recent years regarding learners’ perceived usefulness of OER for learning purposes (Cheung, 2017, 2018, 2019). They addressed the context of university students in Hong Kong, who possess in general an adequate level of readiness for OER (Li and Wong, 2014). A number of characteristics on the students’ perception on the usefulness of OER were identified. For example, the students tend to consider OER more useful for supplementing course textbooks and doing assignments and projects than for preparing tests and examinations. They also tend to consider open courseware, open e-books and online dictionaries more useful than other categories of OER.

The switch of learning mode from typical classroom-based learning to online learning by many higher education institutions during COVID-19 in order to accommodate social distancing requirements provided an opportunity to examine its impact on learners’ perceived usefulness of OER. There have been studies showing that the benefits of using OER for learners in online learning would be different from those in conventional classroom-based learning (Wiley et al., 2017). However, the impact of learning mode on learners’ perceived usefulness of OER has not been systematically studied. Very little is known about how OER are perceived by learners after a rapid change of learning mode caused by the pandemic.

This paper reports the results of a study to address this literature gap. The study involved two identical surveys conducted in 2019 (where classroom-based learning was adopted) and 2021 (where online learning was adopted) participated by university students studying in a face-to-face mode and a distance learning mode in Hong Kong. The surveys focused on students’ perception of OER, which reveal the impact of a rapid switch to online learning for learners’ perceived usefulness of OER. The results during the pandemic period have been found different from those in the previous years. The change in the perceived usefulness occurred for both students studying in the face-to-face mode as well as the distance learning mode. The following research questions are addressed:

- How was learners’ perceived usefulness of OER changed after switching to online learning?
- What are the differences between face-to-face learners and distance learners in their perceived usefulness of OER?

The rest of this paper is structured as follows. Following this introduction, Section 2 presents a review of related studies on the usefulness of OER as well as the impact of learning mode. Section 3 describes the research method, and Section 4 reports the results of this study, which addressed learners’ perceived usefulness of OER for learning purposes as well as concerns about the shortcomings of OER. In Section 5, the results are discussed in relation to those in the related studies. Section 6 concludes this paper, where potential future studies are suggested.

## 2. Literature review

### 2.1. Usefulness of OER

The use of OER has shown various benefits for education. It facilitates faculty members to familiarize themselves with relevant skills and expertise for OER use, as well as enhance knowledge of up-to-date instructional technology developments and technological and pedagogical competencies (Okada et al., 2012; Stagg et al., 2018). Positive evaluations have also been made by university teachers using OER who reported an improvement in course quality, for example in terms of content relevance as they need to carefully screen materials appropriate to the courses being taught (Bliss et al., 2013; Ives and Pringle, 2013).

From the learner perspective, the usefulness of OER has been widely reported. Learners can get access to education more easily, especially for those living in a place where learning opportunities are very limited (Conole, 2012; Beetham, 2013; Bowen et al., 2014). They are also allowed to reduce expenditures on learning materials including textbooks in particular, resulting in a high rate of completion in the courses they take (Hilton and Laman, 2012). Through studying with relevant OER materials, learners may flexibly choose their learning goals from gaining educational qualifications to developing their career (Shank, 2013; Hatzipanagos and Gregson, 2015; Grewe and Davis, 2017). The use of OER in online courses has been observed to encourage differentiated learning by reaching students at their present academic levels (Wiley et al., 2017).

The study of OER usefulness has been commonly related to learners’ perception (Otto et al., 2021). Harsasi (2015) looked at university students’ perceptions of the usefulness of the integration of OER into e-learning, and observed positive comments from the students reporting that using OER facilitates their understanding of the subject knowledge being taught in the lessons and enhances their technological competencies. Lin and Tang (2017) explored how university students perceived the adoption of OER to reduce statistics anxiety in their research methodology courses. They found that the adoption is useful for students to master and make use of research and problem-solving skills in their courses. Other relevant studies include Cooney (2016), Gurung (2017a,b), Jhangiani et al. (2018), and Ocean et al. (2019), where positive perceived usefulness of OER were noted among university students indicating that OER are helpful in improving their learning.

Cheung (2017, 2018, 2019) performed a series of studies to examine how university students, full time and distance learning, perceived the use of OER in their learning. The studies noted high levels of usefulness of OER among both groups who regarded OER as helpful in supplementing course textbooks and materials, acquiring more knowledge as learning reference, and gaining resources for completing assignments and projects and for preparing course assessments. Both groups considered open courseware, open e-books, and open learning software or tools useful for learning. In terms of open online courses and online learning platforms, distance learning students tended to view them as being more useful than full time students did.

### 2.2. Impacts of the COVID-19 pandemic on educational delivery and OER

The outbreak of the COVID-19 pandemic has brought tremendous changes in teaching and learning. A number of studies have been conducted in analyzing how the pandemic influences teaching and learning in various educational contexts in terms broadly of teaching and learning materials and approaches (Chen, 2020; Kanoksilapatham, 2021; Williams and Werth, 2021). With respect to studies into the impacts of COVID-19 on educational delivery, March et al. (2021) noted distinct changes in both the completion rates and the delivery modes of courses during the pandemic. They found, for example, that the completion rates of asynchronous online courses increased a lot more than those of live in-person courses, which may be attributed to the implementation of social distancing during the pandemic. Torda and Shulruf (2021) investigated whether face-to-face and online modes of delivery influenced learning outcomes, social outcomes, and wellbeing among students during the pandemic.

However, the impacts of the pandemic on learners’ perceived usefulness of OER have not been systematically explored in the literature. Very little is known about how OER are perceived by students especially after a rapid change from face-to-face learning to online learning caused by the pandemic crisis. To address this gap, the present study extended the line of prior research in the field of OER by examining how the change in the mode of learning during the pandemic has impacted on the perceived usefulness of OER among university students.

## 3. Research method

This study aimed to examine the perceived usefulness of university students on OER in relation to the change in the learning mode during the COVID-19 pandemic. It covered two groups of students—one studying in a face-to-face mode and another one a distance learning mode. Before the pandemic, the face-to-face students studied in a traditional classroom setting and the distance learning students in a flexible setting including mainly online together with some face-to-face elements. During the pandemic, both groups of students rapidly switched to fully online learning. The study was conducted in the Hong Kong Metropolitan University which offers both face-to-face and distance learning programs.

An online survey was developed for this study. It was conducted twice—one in December 2019 (before the COVID-19 pandemic) and the other in February 2021 (during the pandemic). The participants include face-to-face students and distance learning students of the Hong Kong Metropolitan University. For the survey conducted in 2019, total of 489 and 423 valid responses were received from face-to-face students and distance learning students, respectively. Their average ages are 21.65 years and 32.32 years, respectively. For the other survey conducted in 2021, a total of 624 and 394 valid responses were received from the two groups, respectively. Their average ages are 21.82 and 31.51 years, respectively.

The survey was based on the one used in a series of studies on students’ perceived usefulness of OER (Cheung 2017, 2018, 2019). It contains three sections. The first section collects students’ overall feedback on the usefulness of OER for learning purposes. There are questions, asking students on their agreeance or dis-agreeance to the usefulness of OER for difference learning purposes. The second section focuses on students’ perceived usefulness of various categories of OER. There are questions, asking students on their agreeance or dis-agreeance to the usefulness of different categories of OER. The third section addresses students’ perceived shortcomings and concerns on OER. There are questions, asking students on their agreeance or dis-agreeance to different shortcomings and concerns of OER.

A 5-point Likert scale is adopted for the response to each question, namely, “strongly agree,” “agree,” “neutral,” “disagree,” and “strongly disagree.” The response is converted to a numeric score, where 2 is for “strongly agree,” 1 for “agree,” 0 for “neutral,” −1 for “disagree,” and −2 for “strongly disagree.” For analysis purposes, a weighted score is calculated, as follows.


$$\mathrm{Score}=\left(p_{\mathrm{sa}}\times 2\right)+\left(p_{a}\times 1\right)+\left(p_{n}\times 0\right)+\left(p_{d}\times -1\right)+\left(p_{\mathrm{sd}}\times -2\right).$$

where p_sa_, p_a_, p_n_, p_d,_ and p_sd_ are the percentages of “strongly agree,” “agree,” “neutral,” “disagree,” and “strongly disagree” responses, respectively.

The weighted score is used to interpret the students’ general level of agreeance (or dis-agreeance) in a quantitative scale between −2 and 2. A weighted score closer to 0 (−0.5 to 0.5) means that students are generally neutral to a question. A weighted score more than 0.5 means that students generally agree to a question, and that a higher score implies the stronger agreeance. In contrast, a weight score less than 0.5 means that students generally disagree to a question, and that a lower score implies the stronger dis-agreeance.

## 4. Results

### 4.1. Overall usefulness of OER for learning purposes

Table 1 shows the students’ perception about the overall usefulness of OER for various learning purposes. For each survey, the students’ responses of OER usefulness for each learning purpose are shown together with the difference between the scores from the two surveys. OER were generally perceived to be more useful for supplementing course textbooks and materials and getting resources for doing assignments and projects. Comparing the results of the two surveys, the perceived usefulness of OER increased for all learning purposes in the 2020/2021 survey than that in the 2019/2020 survey.

**Table 1:** Overall usefulness of OER for various learning purposes.

Learning purposes	Face-to-face students’ scores			Distance learning students’ scores		
	2019/2020 (*n* = 489)	2020/2021 (*n* = 624)	Difference	2019/2020 (*n* = 423)	2020/2021 (*n* = 394)	Difference
Supplementing course textbooks and materials	1.06	1.08	0.02	0.96	1.07	0.11
Acquiring more knowledge as learning reference	0.83	1.00	0.17	0.86	0.97	0.11
Getting resources for doing assignments and projects	0.97	1.07	0.10	0.95	1.11	0.16
Getting resources for preparing tests and examination	0.66	0.82	0.16	0.70	0.78	0.08

* A score is calculated as (p_sa_ × 2) + (p_a_ × 1) + (p_n_ × 0) + (p_d_ × −1) + (p_sd_ × −2), where p_sa_, p_a_, p_n_, p_d_, and p_sd_ are the percentages of “strongly agree,” “agree,” “neutral,” “disagree,” and “strongly disagree” responses, respectively.

The result suggests that OER would serve as a more useful support for learning in a fully online mode during the pandemic. For supplementing course textbooks and materials, there is only a slight increase (0.02) in the perceived usefulness from face-to-face students, but a larger increase (0.11) from distance learning students. For getting resources for preparing tests and examination, there is a larger increase (0.16) from face-to-face students but a slight increase (0.08) from distance learning students. The results suggest that the extent of usefulness of OER would depend on the study mode.

### 4.2. Usefulness of specific categories of OER for learning purposes

The second section of the survey covers students’ perceived usefulness of OER by different categories. Table 2 reports students’ perceived usefulness of open courseware and course materials. Between the two surveys, the perceived usefulness of face-to-face students in 2020/2021 is higher than that in 2019/2020 for all types of open courseware and course materials, with a difference of 0.12–0.19 in their scores. However, there is no clear difference in the scores of distance learning students between the two surveys, i.e., −0.02 − 0.08. Open courseware and course materials, in particular supplementary online learning materials, were perceived as more useful by face-to-face students than distance learning students during the switch to online learning.

**Table 2:** Usefulness of various types of open courseware and course materials.

Types of open courseware and course materials	Face-to-face students’ scores^*^			Distance learning students’ scores^*^		
	2019/2020 (*n* = 489)	2020/2021 (*n* = 624)	Difference	2019/2020 (*n* = 423)	2020/2021 (*n* = 394)	Difference
Openly shared complete sets of course materials	1.01	1.13	0.12	1.01	1.09	0.08
Openly shared lecture notes and class notes	0.99	1.13	0.14	1.06	1.07	0.01
Openly shared video clips of lectures and classes	0.95	1.09	0.14	1.02	1.07	0.05
Other supplementary online learning materials	0.86	1.05	0.19	1.00	0.98	−0.02

* A score is calculated as (p_sa_ × 2) + (p_a_ × 1) + (p_n_ × 0) + (p_d_ × −1) + (p_sd_ × −2), where p_sa_, p_a_, p_n_, p_d_, and p_sd_ are the percentages of “strongly agree,” “agree,” “neutral,” “disagree,” and “strongly disagree” responses, respectively.

Table 3 presents the perceived usefulness of open online courses, tutorials and forums. Both the face-to-face students and distance learning students overall indicated a higher level of usefulness for all types of open online courses, tutorials and forums in the 2020/2021 survey. The increase was more prominent for that of face-to-face students on open online self-contained courses as well as open online tutorials on specific topics, with a difference of 0.32 and 0.29 in their scores, respectively. Comparatively, the differences of scores by distance learning students for these two types of OER were 0.11 and 0.13, respectively. Despite such increase, it is worth noting that the perceived usefulness of both groups of students on small-scale mobile learning courses and applications as well as online help desks and forums was at a relatively low level in the 2020/2021 survey, i.e., 0.35–0.54. The two types of OER are relatively not as useful as the others.

**Table 3:** Usefulness of various types of open online courses, tutorials, and forums.

Types of open online courses, tutorials, and forum	Face-to-face students’ scores^*^			Distance learning students’ scores^*^		
	2019/2020 (*n* = 489)	2020/2021 (*n* = 624)	Difference	2019/2020 (*n* = 423)	2020/2021 (*n* = 394)	Difference
Open online courses and self-contained courses	0.66	0.98	0.32	0.94	1.05	0.11
Open online tutorials on specific topics	0.56	0.85	0.29	0.70	0.83	0.13
Small-scale mobile learning courses and applications	0.27	0.35	0.08	0.32	0.40	0.08
Open online interactive help desks and forums	0.23	0.39	0.16	0.44	0.54	0.10

* A score is calculated as (p_sa_ × 2) + (p_a_ × 1) + (p_n_ × 0) + (p_d_ × −1) + (p_sd_ × −2), where p_sa_, p_a_, p_n_, p_d_, and p_sd_ are the percentages of “strongly agree,” “agree,” “neutral,” “disagree,” and “strongly disagree” responses, respectively.

Table 4 reports the perceived usefulness of open access e-books, journals, reports and other documents. There was an increase in the perceived usefulness for all of these in the 2020/2021 survey for both groups of students. The increase was particularly prominent for face-to-face students for open access e-books which include both self-contained textbooks and reference books, with a difference of 0.32 and 0.29 in scores for these two types, respectively. The result suggests that open access e-books would be relatively more helpful for face-to-face students for learning in an online mode.

**Table 4:** Usefulness of various types of open access e-books, journals, reports, and other documents.

Types of open access books, journals, and other documents	Face-to-face students’ scores^*^			Distance learning students’ scores^*^		
	2019/2020 (*n* = 489)	2020/2021 (*n* = 624)	Difference	2019/2020 (*n* = 423)	2020/2021 (*n* = 394)	Difference
Open access e-books (self-contained textbooks)	0.79	1.11	0.32	1.10	1.26	0.16
Open access e-books (self-contained reference books)	0.75	1.04	0.29	1.05	1.19	0.14
Open access journals, magazines and periodicals	0.58	0.74	0.16	0.64	0.80	0.16
Open access reports and other documents	0.78	0.93	0.15	0.78	0.90	0.12

* A score is calculated as (p_sa_ × 2) + (p_a_ × 1) + (p_n_ × 0) + (p_d_ × −1) + (p_sd_ × −2), where p_sa_, p_a_, p_n_, p_d_, and p_sd_ are the percentages of “strongly agree,” “agree,” “neutral,” “disagree,” and “strongly disagree” responses, respectively.

Table 5 reports the perceived usefulness of open source learning software, tools and platforms. All of these types of OER were perceived by distance learning students as more useful in 2020/2021. For face-to-face students, only online anti-plagiarism checker and grammar checker as well as online learning platform for self and collaborative learning were perceived as more useful in 2020/2021. There was no clear change in their perceived usefulness for open online dictionaries and encyclopedia as well as online learning software, with differences of −0.07 and 0.02 in scores between the 2 years for these two types, respectively.

**Table 5:** Usefulness of various types of open source learning software, tools, and platforms.

Types of open source learning software and tools	Face-to-face students’ scores^*^			Distance learning students’ scores^*^		
	2019/2020 (*n* = 489)	2020/2021 (*n* = 624)	Difference	2019/2020 (*n* = 423)	2020/2021 (*n* = 394)	Difference
Open online dictionaries and encyclopedia	1.00	0.93	−0.07	0.96	1.07	0.11
Online anti-plagiarism checker and grammar checker	0.97	1.07	0.10	0.79	0.92	0.13
Online learning software (mind-map, slide-builder, etc.)	0.84	0.86	0.02	0.62	0.76	0.14
Online platform for self and collaborative learning	0.72	0.82	0.10	0.74	0.85	0.11

* A score is calculated as (p_sa_ × 2) + (p_a_ × 1) + (p_n_ × 0) + (p_d_ × −1) + (p_sd_ × −2), where p_sa_, p_a_, p_n_, p_d_, and p_sd_ are the percentages of “strongly agree,” “agree,” “neutral,” “disagree,” and “strongly disagree” responses, respectively.

### 4.3. Concerns about the shortcomings of OER

Like many open online resources on the Internet, OER also have shortcomings such as inaccurate, irrelevant and incomplete contents. The accuracy, readability, completeness, comprehensiveness and relevancy of the OER contents are students’ major concerns (Tang, 2020). Some OER providers have established self-regulatory guidelines or measures for assuring quality of the contents before publishing on the Internet. These are usually found in open access textbook platforms, where quality assurance is essentially required (Cheung et al., 2015; Belikov and McLure, 2020; Cheung, 2020).

Table 6 reports the students’ concerns about the shortcomings of OER for learning purposes, covering the accuracy, updatedness, comprehensiveness and organization of OER contents. For both surveys, the students’ concerns were rather neutral. Both face-to-face and distance learning students had relatively more concerns about the accuracy and comprehensiveness of the contents. In the 2020/2021 survey, the extent of concerns of face-to-face students was slightly higher for the accuracy and updatedness of OER contents, while distance learning students showed less concern for the updatedness and comprehensiveness of OER contents.

**Table 6:** Concerns about OER for learning purposes.

Concerns about OER	Face-to-face students’ scores^*^			Distance learning students’ scores^*^		
	2019/2020 (*n* = 489)	2020/2021 (*n* = 624)	Difference	2019/2020 (*n* = 423)	2020/2021 (*n* = 394)	Difference
Some contents may not be accurate	0.30	0.48	0.18	0.39	0.34	−0.05
Some contents may not be up-to-date	0.01	0.20	0.19	0.28	0.14	−0.14
Some contents may not be comprehensive	0.29	0.39	0.10	0.43	0.31	−0.12
Some contents may not be well organized	0.17	0.19	0.02	0.21	0.24	0.03

* A score is calculated as (p_sa_ × 2) + (p_a_ × 1) + (p_n_ × 0) + (p_d_ × −1) + (p_sd_ × −2), where p_sa_, p_a_, p_n_, p_d_, and p_sd_ are the percentages of “strongly agree,” “agree,” “neutral,” “disagree,” and “strongly disagree” responses, respectively.

## 5. Discussion

For many years, OER have been used for different learning purposes and their usefulness has been widely studied (Ocean et al., 2019; Otto et al., 2021). As a response to the social distancing measures since the outbreak of the pandemic, the switch of teaching and learning mode by higher education institutions in a short span of time from their usual classroom-based to fully online learning has posed an unprecedented challenge in ensuring the continuity of teaching and learning while maintaining teaching quality and learning effectiveness. Not only face-to-face students, the change in teaching mode has also impacted distance learning students. With the features of free and open availability on the Internet, OER have served as a suitable and timely support for both groups of students for their learning during the pandemic.

Overall positive feedback from the students on the usefulness of OER during the pandemic has been shown in this study. Both groups of the students showed an increase in their perceived usefulness of OER in terms of getting more reference materials for learning, doing assignments and projects. This finding is consistent with related studies, where OER allows learners to get access to educational materials more easily (Beetham, 2013; Bowen et al., 2014). Also, learners may flexibly select suitable materials to cope with their learning goals (Hatzipanagos and Gregson, 2015; Grewe and Davis, 2017).

There are also differences in the change of perceived usefulness between face-to-face and distance learning students in online learning. Face-to-face students indicated a relatively higher extent of perceived usefulness for using OER for preparing tests and examination during the pandemic, while distance learning students revealed a higher extent for using OER for supplementing course textbooks and materials. The results may reflect their changes in learning process, learning styles and habits, and also the changes in their usage of OER as well as their perception of the usefulness of OER (Wong and Li, 2020; Wong and Wong, 2020). Following the findings in Wiley et al. (2017) that the use of OER in online courses encouraged differentiated learning, the results on the differences in perceived usefulness between face-to-face and distance learning students suggest the need to recognize the diverse needs of these two groups of students for tailoring learning activities for them.

The diverse needs of face-to-face and distance learning students are more clearly shown in their perceived usefulness of specific types of OER. Face-to-face students perceived open courseware and course materials as more useful during the pandemic, while distance learning students did not show an observable change in perceived usefulness of these types of OER. This would be because the distance learning students were usually already provided with a complete set of course materials including also online references for their self-study (Harsasi, 2015; Cheung, 2018), thus their demand for additional course materials may not be as large as face-to-face students after switching to online learning. On the other hand, both groups of students showed a relatively high level of perceived usefulness for resources such as open access textbooks, reference books, and online courses. This suggests that the quality of these resources was recognized by the students which is also observed in related studies (Gurung, 2017a,b; Jhangiani et al., 2018). Also, the students may need more instructional support during the fully online teaching as reflected from their need for the resources.

As explained in Cheung (2021), many higher education institutions responded to the pandemic through delivering lectures and tutorials online *via* video-conferencing tools, on the same duration of lessons and without any change in the contents. Learning effectiveness would be affected by just a switch on the delivery mode without any changes on the contents and forms. The needs of students for various types of OER imply potential problems in teaching and learning they faced during the fully online learning, and faculty members should tailor their teaching to cope with students’ needs for learning in the online mode (Wong et al., 2021).

The students’ concerns about OER for learning purposes remained at a relatively low level in both surveys. This result is consistent with their overall positive feedback on the perceived usefulness of OER, which has been also reported in related studies (Cheung, 2017, 2018, 2019). There are differences between face-to-face and distance learning students in their concerns shown in the two surveys. The face-to-face students revealed a slight increase in the extent of concerns about the accuracy, updatedness and comprehensiveness of OER contents, while the distance learning students showed a slight decrease in the extent of these concerns. The differences would be related to changes in their learning needs and experience in OER for learning during the pandemic period. It has been raised in relevant studies that learners’ perceived difficulties in relation to OER use during the COVID-19 pandemic are related to the familiarity (Chen, 2020) and competence of working with the resources (Huang et al., 2020).

## 6. Conclusion

This paper reports the usefulness of OER perceived by university students after a rapid switch to online learning during the pandemic period. The findings contribute to revealing the needs of learners for OER in relation to the change of learning mode. They show that the students generally perceived OER to be more useful during their learning in online mode, in particular for supplementing course textbooks and materials, and getting resources for doing assignments and projects. The specific types of OER which were perceived as relatively more useful include openly shared course materials, open online courses, open access e-textbooks, open online dictionaries, anti-plagiarism checkers and grammar checkers. On the other hand, the students concerned about the shortcomings of OER, especially on the accuracy and comprehensiveness of the contents. The findings suggest that students’ perceived usefulness of OER would be relevant to their learning mode. These findings supplement relevant literature with other focuses such as advocacy of OER use in response to the pandemic (Huang et al., 2020; Lee and Lee, 2021) and development of related resources (Chen, 2020).

This paper also addresses the diverse needs of face-to-face students and distance learning students. The results show that face-to-face students perceived as more useful resources such as open courseware and course materials, while distance learning students did not show an observable change in perceived usefulness of these resources after switching to online learning. Their needs for various types of resources reveal potential problems they faced during online learning. The results thus facilitate faculty members to tailor their teaching to cope with students’ diverse needs for learning in the online mode.

The limitation of this study should be noted. The surveys in this study focused on the perspective of university students in Hong Kong. For a comprehensive understanding of the usefulness of OER, future work should address other regions where learners’ needs for OER may be different. Also, the perspectives of other stakeholders such as faculty members should be covered.

The results of this study open up opportunities for future work to examine how OER support can be tailored for various groups of students. The differences in perceived usefulness of OER between face-to-face and distance learning students suggest future studies to examine their diverse needs for learning resources as well as instructional support for tailoring learning activities for them. Besides, it can be envisaged that students’ learning mode will be switched again after the pandemic, be it back to conventional face-to-face classroom teaching or a hybrid mode. Students’ needs for and perceived usefulness of OER may change again at that time. Future studies should address more on how this factor affects students’ perceptions and actual use of OER.

## Data availability statement

The raw data supporting the conclusions of this article will be made available by the authors, without undue reservation.

## Author contributions

All authors listed have made a substantial, direct, and intellectual contribution to the work and approved it for publication.

## Conflict of interest

The authors declare that the research was conducted in the absence of any commercial or financial relationships that could be construed as a potential conflict of interest.

## Publisher’s note

All claims expressed in this article are solely those of the authors and do not necessarily represent those of their affiliated organizations, or those of the publisher, the editors and the reviewers. Any product that may be evaluated in this article, or claim that may be made by its manufacturer, is not guaranteed or endorsed by the publisher.
