# Supplementary material for: Perceived usefulness of open educational resources: Impact of switching to online learning for face-to-face and distance learners
Source: Front Psychol. 2023 Jan 18;13:1004459. doi: 10.3389/fpsyg.2022.1004459 (PMC9891131; doi:10.3389/fpsyg.2022.1004459)
Supplement: Supplementary file 1 [file Data_Sheet_1.PDF]

Appendix 1: Survey on the Perceived Usefulness of OER (Full-time Students) in 2019-20

Number of responses : 489

Overall usefulness of OER for various learning purposes

Learning Purposes	Strongly Agree	Agree	Neutral	Disagree	Strongly Disagree
Supplementing course textbooks and materials	40%	36%	17%	4%	3%
Acquiring more knowledge as learning reference	33%	31%	26%	6%	4%
Getting resources for doing assignments and projects	37%	37%	17%	5%	3%
Getting resources for preparing tests and examination	28%	33%	24%	8%	6%

Usefulness of various types of open courseware and course materials

Types of Open Courseware and Course Materials	Strongly Agree	Agree	Neutral	Disagree	Strongly Disagree
Openly shared complete sets of course materials	41%	31%	20%	4%	4%
Openly shared lecture notes and class notes	42%	28%	21%	5%	4%
Openly shared video clips of lectures and classes	39%	27%	25%	7%	3%
Other supplementary online learning materials	34%	31%	26%	5%	4%

Usefulness of various types of open online courses, tutorials and forums

Types of Open Online Courses, Tutorials and Forum	Strongly Agree	Agree	Neutral	Disagree	Strongly Disagree
Open online courses and self-contained courses	31%	26%	27%	9%	8%
Open online tutorials on specific topics	23%	32%	29%	10%	6%
Small-scale mobile learning courses and applications	19%	21%	36%	15%	10%
Open online interactive help desks and forums	20%	21%	31%	18%	10%

Usefulness of various types of open access e-books, journals, reports and other documents

Types of Open Access Books, Journals and other Documents	Strongly Agree	Agree	Neutral	Disagree	Strongly Disagree
Open access e-books (self-contained textbooks)	37%	26%	21%	11%	5%
Open access e-books (self-contained reference books)	35%	27%	22%	11%	4%
Open access journals, magazines and periodicals	30%	23%	26%	16%	6%
Open access reports and other documents	34%	30%	21%	10%	5%

Usefulness of various types of open source learning software, tools and platforms

Types of Open Source Learning Software and Tools	Strongly Agree	Agree	Neutral	Disagree	Strongly Disagree
Open online dictionaries and encyclopaedia	39%	33%	19%	6%	4%
Online anti-plagiarism checker and grammar checker	40%	30%	21%	5%	4%
Online learning software (mind-map, slide-builder, etc.)	31%	33%	27%	6%	4%
Online platform for self and collaborative learning	30%	30%	27%	8%	5%

Concerns about OER for learning purposes

Concerns about OER	Strongly Agree	Agree	Neutral	Disagree	Strongly Disagree
Some contents may not be accurate	16%	31%	27%	19%	7%
Some contents may not be up-to-date	10%	22%	37%	21%	10%
Some contents may not be comprehensive	15%	29%	33%	16%	7%
Some contents may not be well organised	15%	23%	34%	19%	10%

Appendix 2: Survey on the Perceived Usefulness of OER (Full-time Students) in 2020-20

Number of responses : 624

Overall usefulness of OER for various learning purposes

Learning Purposes	Strongly Agree	Agree	Neutral	Disagree	Strongly Disagree
Supplementing course textbooks and materials	38%	39%	19%	2%	1%
Acquiring more knowledge as learning reference	36%	38%	19%	5%	1%
Getting resources for doing assignments and projects	40%	36%	17%	5%	2%
Getting resources for preparing tests and examination	34%	31%	23%	8%	3%

Usefulness of various types of open courseware and course materials

Types of Open Courseware and Course Materials	Strongly Agree	Agree	Neutral	Disagree	Strongly Disagree
Openly shared complete sets of course materials	43%	32%	20%	4%	2%
Openly shared lecture notes and class notes	45%	31%	18%	4%	2%
Openly shared video clips of lectures and classes	44%	31%	18%	5%	1%
Other supplementary online learning materials	39%	33%	22%	5%	2%

Usefulness of various types of open online courses, tutorials and forums

Types of Open Online Courses, Tutorials and Forum	Strongly Agree	Agree	Neutral	Disagree	Strongly Disagree
Open online courses and self-contained courses	35%	37%	21%	5%	2%
Open online tutorials on specific topics	28%	36%	28%	6%	2%
Small-scale mobile learning courses and applications	19%	25%	36%	13%	6%
Open online interactive help desks and forums	22%	25%	32%	12%	9%

Usefulness of various types of open access e-books, journals, reports and other documents

Types of Open Access Books, Journals and other Documents	Strongly Agree	Agree	Neutral	Disagree	Strongly Disagree
Open access e-books (self-contained textbooks)	46%	27%	20%	5%	3%
Open access e-books (self-contained reference books)	44%	28%	19%	6%	3%
Open access journals, magazines and periodicals	36%	23%	25%	11%	5%
Open access reports and other documents	41%	26%	22%	7%	4%

Usefulness of various types of open source learning software, tools and platforms

Types of Open Source Learning Software and Tools	Strongly Agree	Agree	Neutral	Disagree	Strongly Disagree
Open online dictionaries and encyclopaedia	37%	32%	20%	8%	4%
Online anti-plagiarism checker and grammar checker	40%	34%	20%	5%	1%
Online learning software (mind-map, slide-builder, etc.)	35%	31%	23%	7%	4%
Online platform for self and collaborative learning	32%	32%	25%	8%	3%

Concerns about OER for learning purposes

Concerns about OER	Strongly Agree	Agree	Neutral	Disagree	Strongly Disagree
Some contents may not be accurate	20%	31%	31%	13%	5%
Some contents may not be up-to-date	12%	29%	33%	19%	7%
Some contents may not be comprehensive	17%	32%	30%	15%	6%
Some contents may not be well organised	14%	26%	33%	19%	8%

<u>Appendix 3: Survey on the Perceived Usefulness of OER (Distance-learning Students) in 2019-20</u>

Number of responses : 423

Overall usefulness of OER for various learning purposes

Learning Purposes	Strongly Agree	Agree	Neutral	Disagree	Strongly Disagree
Supplementing course textbooks and materials	34%	39%	20%	6%	2%
Acquiring more knowledge as learning reference	32%	35%	25%	6%	3%
Getting resources for doing assignments and projects	35%	37%	19%	8%	1%
Getting resources for preparing tests and examination	29%	33%	24%	9%	5%

Usefulness of various types of open courseware and course materials

Types of Open Courseware and Course Materials	Strongly Agree	Agree	Neutral	Disagree	Strongly Disagree
Openly shared complete sets of course materials	42%	29%	23%	4%	3%
Openly shared lecture notes and class notes	42%	33%	19%	3%	3%
Openly shared video clips of lectures and classes	40%	32%	21%	4%	3%
Other supplementary online learning materials	38%	33%	20%	4%	4%

Usefulness of various types of open online courses, tutorials and forums

Types of Open Online Courses, Tutorials and Forum	Strongly Agree	Agree	Neutral	Disagree	Strongly Disagree
Open online courses and self-contained courses	38%	32%	21%	5%	5%
Open online tutorials on specific topics	26%	34%	30%	5%	5%
Small-scale mobile learning courses and applications	20%	22%	37%	11%	10%
Open online interactive help desks and forums	21%	29%	30%	11%	8%

Usefulness of various types of open access e-books, journals, reports and other documents

Types of Open Access Books, Journals and other Documents	Strongly Agree	Agree	Neutral	Disagree	Strongly Disagree
Open access e-books (self-contained textbooks)	46%	31%	15%	5%	4%
Open access e-books (self-contained reference books)	44%	31%	17%	6%	3%
Open access journals, magazines and periodicals	31%	25%	27%	11%	5%
Open access reports and other documents	36%	27%	25%	8%	5%

Usefulness of various types of open source learning software, tools and platforms

Types of Open Source Learning Software and Tools	Strongly Agree	Agree	Neutral	Disagree	Strongly Disagree
Open online dictionaries and encyclopaedia	38%	30%	24%	5%	2%
Online anti-plagiarism checker and grammar checker	32%	32%	25%	6%	5%
Online learning software (mind-map, slide-builder, etc.)	24%	32%	30%	11%	3%
Online platform for self and collaborative learning	29%	33%	27%	7%	4%

Concerns about OER for learning purposes

Concerns about OER	Strongly Agree	Agree	Neutral	Disagree	Strongly Disagree
Some contents may not be accurate	16%	30%	36%	14%	5%
Some contents may not be up-to-date	12%	31%	37%	16%	5%
Some contents may not be comprehensive	17%	31%	34%	14%	4%
Some contents may not be well organised	13%	23%	41%	18%	5%

<u>Appendix 4: Survey on the Perceived Usefulness of OER (Distance-learning Students) in 2020-21</u>

Number of responses : 394

Overall usefulness of OER for various learning purposes

**Learning Purposes**	**Strongly Agree**	**Agree**	**Neutral**	**Disagree**	**Strongly Disagree**
Supplementing course textbooks and materials	37%	43%	15%	3%	3%
Acquiring more knowledge as learning reference	34%	39%	21%	4%	2%
Getting resources for doing assignments and projects	41%	38%	15%	4%	2%
Getting resources for preparing tests and examination	33%	34%	19%	9%	5%

Usefulness of various types of open courseware and course materials

**Types of Open Courseware and Course Materials**	**Strongly Agree**	**Agree**	**Neutral**	**Disagree**	**Strongly Disagree**
Openly shared complete sets of course materials	44%	30%	22%	3%	2%
Openly shared lecture notes and class notes	41%	36%	19%	3%	3%
Openly shared video clips of lectures and classes	44%	32%	17%	4%	3%
Other supplementary online learning materials	40%	31%	23%	4%	3%

Usefulness of various types of open online courses, tutorials and forums

**Types of Open Online Courses, Tutorials and Forum**	**Strongly Agree**	**Agree**	**Neutral**	**Disagree**	**Strongly Disagree**
Open online courses and self-contained courses	40%	37%	17%	4%	3%
Open online tutorials on specific topics	30%	37%	26%	4%	4%
Small-scale mobile learning courses and applications	21%	26%	34%	11%	8%
Open online interactive help desks and forums	25%	27%	31%	11%	6%

Usefulness of various types of open access e-books, journals, reports and other documents

Types of Open Access Books, Journals and other Documents	Strongly Agree	Agree	Neutral	Disagree	Strongly Disagree
Open access e-books (self-contained textbooks)	54%	28%	13%	3%	3%
Open access e-books (self-contained reference books)	51%	30%	12%	4%	3%
Open access journals, magazines and periodicals	38%	24%	23%	9%	5%
Open access reports and other documents	42%	25%	21%	8%	4%

Usefulness of various types of open source learning software, tools and platforms

Types of Open Source Learning Software and Tools	Strongly Agree	Agree	Neutral	Disagree	Strongly Disagree
Open online dictionaries and encyclopaedia	43%	31%	18%	4%	3%
Online anti-plagiarism checker and grammar checker	38%	32%	20%	5%	4%
Online learning software (mind-map, slide-builder, etc.)	32%	31%	26%	6%	5%
Online platform for self and collaborative learning	36%	33%	21%	6%	6%

Concerns about OER for learning purposes

Concerns about OER	Strongly Agree	Agree	Neutral	Disagree	Strongly Disagree
Some contents may not be accurate	15%	31%	33%	15%	6%
Some contents may not be up-to-date	10%	26%	37%	21%	5%
Some contents may not be comprehensive	13%	31%	33%	18%	4%
Some contents may not be well organised	15%	25%	36%	19%	5%
